# Similarity-Based Modeling Applied to Signal Detection in Pharmacovigilance

**DOI:** 10.1038/psp.2014.35

**Published:** 2014-09-24

**Authors:** S Vilar, P B Ryan, D Madigan, P E Stang, M J Schuemie, C Friedman, N P Tatonetti, G Hripcsak

**Affiliations:** 1 Department of Biomedical Informatics, Columbia University, New York, New York, USA; 2 Observational Health Data Sciences and Informatics (OHDSI), New York, New York, USA; 3 Janssen Research and Development, Titusville, New Jersey, USA; 4 Department of Statistics, Columbia University, New York, New York, USA; 5 Department of Systems Biology, Columbia University Medical Center, New York, New York, USA; 6 Department of Medicine, Columbia University Medical Center, New York, New York, USA

## Abstract

One of the main objectives in pharmacovigilance is the detection of adverse drug events (ADEs) through mining of healthcare databases, such as electronic health records or administrative claims data. Although different approaches have been shown to be of great value, research is still focusing on the enhancement of signal detection to gain efficiency in further assessment and follow-up. We applied similarity-based modeling techniques, using 2D and 3D molecular structure, ADE, target, and ATC (anatomical therapeutic chemical) similarity measures, to the candidate associations selected previously in a medication-wide association study for four ADE outcomes. Our results showed an improvement in the precision when we ranked the subset of ADE candidates using similarity scorings. This method is simple, useful to strengthen or prioritize signals generated from healthcare databases, and facilitates ADE detection through the identification of the most similar drugs for which ADE information is available.

The detection of adverse drug events (ADEs) is a major challenge in pharmacovigilance. With the explosion of electronic data, there has been an increase of availability of different data sources, such as the electronic health records^1^ or administrative claims data,^2^ that have supplemented the existing US Food and Drug Administration Adverse Event Reporting System^3^ of voluntary reports. Analysis of these healthcare databases provides potential opportunity to detect and control the impact of potential adverse effects in the population. However, signal detection methods are still a great challenge in the drug safety scientific community.

Different methodologies have been developed to infer safety signals in healthcare databases between drugs and potential adverse events that merit further investigation.^4,5^ Although encouraging results in ADE detection have been reported for some methods,^6^ some challenges and limitations remain most prominent among which is controlling for potential confounding factors.^5,7^ In fact, the class of approaches taking into account cofounding effects showed better predictive results in an assessment of different signal-detection algorithms in US Food and Drug Administration Adverse Event Reporting System.^8^ Some approaches, such as multiple logistic regression, could be helpful to control cofounding for co-medications in spontaneous reporting systems.^9,10^ Other data sources, such as large-scale observational healthcare data, also provide important information for drug safety, but it is still necessary to determine suitable statistical methods for its analysis.^11,12,13,14^ As an example, disproportionality methods, commonly used in the analysis of spontaneous reports, showed poor performance using observational healthcare data.^15^ However, self-controlled cohort methods showed potential for risk identification in observational databases.^16^ Integration of different sources of information, such as electronic health records and medical literature, is an option that can offer good results in the generation of improved surveillance systems.^17,18,19^

The results obtained by our group in a previous medication-wide association study using an observational healthcare database, the Truven MarketScan Commercial Claims and Encounters (CCAE) database,^2^ showed that most of the signals generated from four clinically important ADE outcomes were positive controls.^12,20^ However, we observed some statistical associations with values above two statistical thresholds, *P* < 0.05 and *P* < 0.0005 (Bonferroni correction), between the ADEs and medications not known to be culpable (false positives). Our previous system,^20^ although capable of generating an enriched subset of ADE candidates, showed some limitations in its ability to exclude some false positives from the final signal selection. Other studies using other data algorithms and data sources showed the potential to provide an enriched set of drug candidates that can cause the ADE.^21,22^ However, improvement in the precision in this enriched set of candidates was achieved through the application of 2D structural similarity.^21,22^

We propose here a complementary method which is based on similarity modeling to rank the subset of ADE candidates generated through self-controlled case series (SCCS) data analysis. Our aim is to improve the precision of the technique in different top candidate positions and consequently provide an efficient way to prioritize signals. **Figure 1** shows the general steps in the development of our analysis.

## Results

### Performance of SCCS analysis

**Figure 2:**
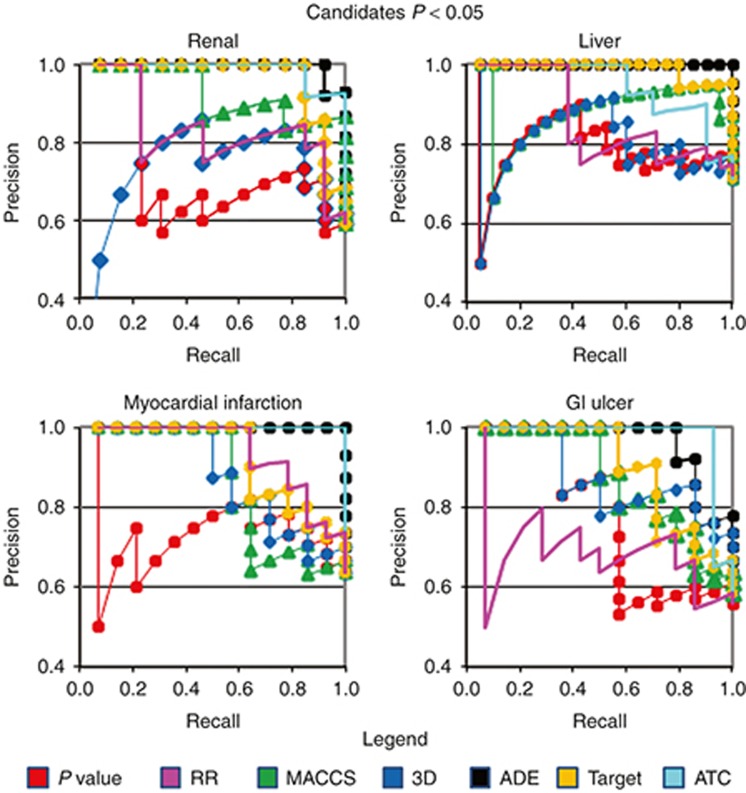
Precision–Recall curves evaluating the four subsets of ADE candidates originated with SCCS analysis (threshold *P* < 0.05) using different methods to rank the candidates (methods: *P* values, relative risk (RR), 2D MACCS, 3D structure similarity, ADE, Target, and ATC similarity). SCCS analysis is useful to originate and select the subset of ADE candidate drugs. Application of similarity-based methods in this selected subset can enrich the precision in different top positions compared with *P* values and RR. For clarity, precision is plotted above 0.4. In the recall calculation, only the drugs in the selected set of drug candidates (*P* < 0.05) were taken into account. In the sets of liver failure and gastrointestinal (GI) ulcer, there are two candidates (the drug–protein candidates interferon beta 1a and lipase, respectively) that cannot be evaluated using similarity-based models.

We applied the SCCS method to the CCAE administrative claims database^2,12^ to estimate associations between sets of drugs defined as positive and negative controls and four diverse ADEs: acute renal failure, acute liver failure, acute myocardial infarction, and upper gastrointestinal (GI) ulcer.^20^ **Table 1** shows sensitivity, specificity, and precision for each ADE. We also calculated the area under the receiver operating characteristic curve (AUROC) for each ADE using *P* values and relative risk (RR) to rank all the drugs in each reference standard. AUROCs range from 0.58 to 0.78. The results showed the capacity of the SCCS analysis to discriminate between drugs that were or were not believed to be causally related to the ADEs. However, within the sets of drugs selected above the two *P*-value thresholds (*P* < 0.05 and *P* < 0.0005), the precision in some cases did not show a clear improvement as the *P* value decreases (**Figure 2** and **Supplementary Figure S1**). As an example, precision in the ADE acute renal failure using the *P-*value scoring method is similar in the top 10 position and the top 20 (precision = 0.6). **Table 2** also shows the area under the ROC curve for the subset of ADE candidates whose *P* values are <0.05 (this partial AUROC is defined in the article as pAUROC). On the other hand, ranking the same final subset of ADE candidates using RR offered better precision results (**Figure 2**) and pAUROCs (**Table 2**). However, as it is explained in the next section, combination of SCCS and similarity methods showed an improvement in ADE detection.

### Performance of similarity-based methods in the SCCS analysis candidates

We applied our 2D and 3D molecular structure, ADE, Target, and ATC similarity-based models to the subset of candidates previously selected through SCCS analysis. We used two different subsets of drug candidates for each ADE based on two different *P*-value thresholds (*P* < 0.05 and *P* < 0.0005). Similarity score assignment for each drug in every ADE dataset was performed taking out one at a time (leave-one-out method) and comparing the similarity against the positive and negative controls in the reference standard (see Methods section). When the drug candidates within the threshold *P* < 0.05 were ranked according to similarity scores, the analysis showed a clear improvement in precision in different top positions as compared with *P* values (**Figure 2** and **Supplementary Table S1**). Using similarity-based methods resulted in greater pAUROCs for the subset of candidates (**Table 2**). When performances of the similarity methods and RR algorithm are compared, similarity methods still show better performance in some of the studied ADE outcomes (**Figure 2** and **Table 2**). We found similar results for the set of candidates selected with the threshold *P* < 0.0005 (see **Supplementary Table S1 and Figure S1**). The use of similarity-based models allowed us to obtain better positive predictive values in some sets. The method is an alternative system to organize the set of ADE candidates with value in better understanding the detected ADE–drug relationships.

### Rationalization of the signals detected in healthcare data

We can identify for each drug in the test the most similar drug in the ADE reference standard, with the consequent associated ADE information available from the literature. This feature facilitates the decision making in the ADE signal evaluation process. In some cases, depending on the available literature, the system could help to generate further hypotheses about possible ADE mechanisms of action. Moreover, these types of models can detect drug pair similarities based on whether both drugs are in the same or different pharmacological classes. Most frequently, the drugs are in the same pharmacological class and the information provided by the system is obvious. When the drugs belong to different classes, the situation becomes more challenging because the drug pair relationship is less apparent.

**Figure 3:**
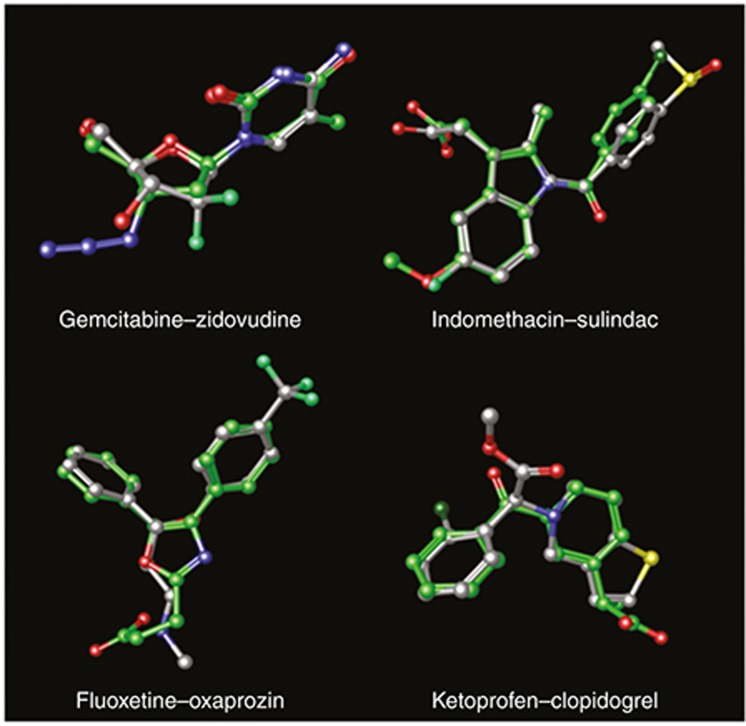
Examples of some drug pairs retrieved by the 3D molecular structure similarity models for the different ADE outcomes. In each pair, a drug is in the leave-one-out test and the other drug is the most similar drug in the ADE reference standard (positive controls). Carbon atoms in each pair are represented in gray and green.

**Figure 3** shows some examples of drugs detected by similarity modeling along with the drug source in the reference standard. Gemcitabine and zidovudine are both nucleoside analogs with clear structural analogy (**Figure 3**). The 3D model associated both drugs with high score (3D_score = 0.84). However, this is a case where both drugs belong to the same structural category but they could be deemed in different pharmacological classes. Gemcitabine is used in clinic to treat different types of cancer, and zidovudine is an antiretroviral agent for the treatment of HIV infection. Both drugs have the potential to cause liver failure, although it is not clear whether they follow similar etiology. Gemcitabine could cause a direct hepatic toxicity, and zidovudine could lead to mitochondrial dysfunction.^23^ Another example of similarity detected by the model is the pair indomethacin–sulindac (3D_score = 0.81). In this case, both drugs are nonsteroidal anti-inflammatory drugs (NSAIDs), belong to the same pharmacological category, and have the potential to cause GI ulcer, liver failure, and acute myocardial infarction.

A pair of drugs belonging to different pharmacological classes and pointed out by the GI ulcer 3D models is fluoxetine–oxaprozin (positive control 3D_score = 0.78). There are some reports that indicate that the antidepressant fluoxetine can have some anti-inflammatory properties,^24^ similar to the NSAID oxaprozin. However, the mechanism of action in both drugs could be different. Oxaprozin is believed to inhibit the enzyme cyclooxygenase (COX) with the consequent inhibition in the synthesis of prostaglandins. Fluoxetine has been reported to inhibit the signaling of toll-like receptors, providing a potential mechanism for their anti-inflammatory action.^24^ The ADE GI ulcer in both drugs could be related to an increased risk of bleeding. Some selective serotonin reuptake inhibitors like fluoxetine have been related to abnormal bleeding. The possible mechanisms for the ADE could be by blocking the uptake of serotonin into platelets causing platelet dysfunction or by an increase in gastric acid secretion leading to a higher risk of GI bleeding.^25^ Although different mechanisms could be implicated in NSAID-induced GI ulcer,^26,27^ platelet inhibition caused by NSAIDs could also be an important factor to explain an increased risk of bleeding and therefore GI ulcer.^28^

The 3D molecular structure model in GI ulcer also detected that clopidogrel, an antiplatelet agent, is similar to the NSAID ketoprofen (positive control 3D_score = 0.79). Both drugs have a different mechanism of action because clopidogrel is an inhibitor of the P2Y_12_ adenosine diphosphate receptor and ketoprofen develops the anti-inflammatory activity through the inhibition of the COX enzyme.^29^ However, both drugs share some pharmacological actions. Both mechanisms, adenosine diphosphate and COX inhibition, are implicated in the inactivation of platelets. There is available information that relates some NSAIDs with antiplatelet properties, i.e., the antiplatelet properties of ketoprofen or acetylsalicylic acid are known.^30,31^ On the other hand, clopidogrel has been reported to provide anti-inflammatory renoprotective effects, although the mechanism of action is still unclear.^32^ Both drugs can cause upper GI ulceration, probably accentuated because of the ADE bleeding associated with their antiplatelet properties. However, the mechanism of action by which clopidogrel and ketoprofen cause GI ulcer is also probably different. Although clopidogrel's mechanism remains unclear, clopidogrel does not have an effect on the COX pathway.^33^ On the other hand, ketoprofen is believed to cause the ADE GI ulcer due to the inhibition of COX-1.^29^ Moreover, NSAIDs can cause damage in the GI mucosa following other mechanisms, such as topical irritating effects on the epithelium or blood flow reduction in the mucosa.^27^ Although it is hard to reconcile the different mechanisms of action with the high 3D similarity, it is worth noting that we also found that clopidogrel is similar to other NSAIDs, such as etodolac (0.76), bromfenac (0.74), tolmetin (0.73), ketorolac (0.73), mefenamate (0.73), valdecoxib (0.72), fenoprofen (0.72), and flurbiprofen (0.72). In fact, in the GI ulcer reference standard, there are nine NSAIDs within the most 15 similar drugs to clopidogrel according to the 3D score. However, similarity is not high with all the NSAIDs. For instance, 3D similarity score between clopidogrel and acetylsalicylic acid is 0.51. The association showed by the model does not provide enough evidence that these drugs cause the ADE GI ulcer through the same mechanism of action, and more studies would be necessary to detect if this association is true.

### Visualizing correlations between similarity measures and pharmacological classes

**Figure 4:**
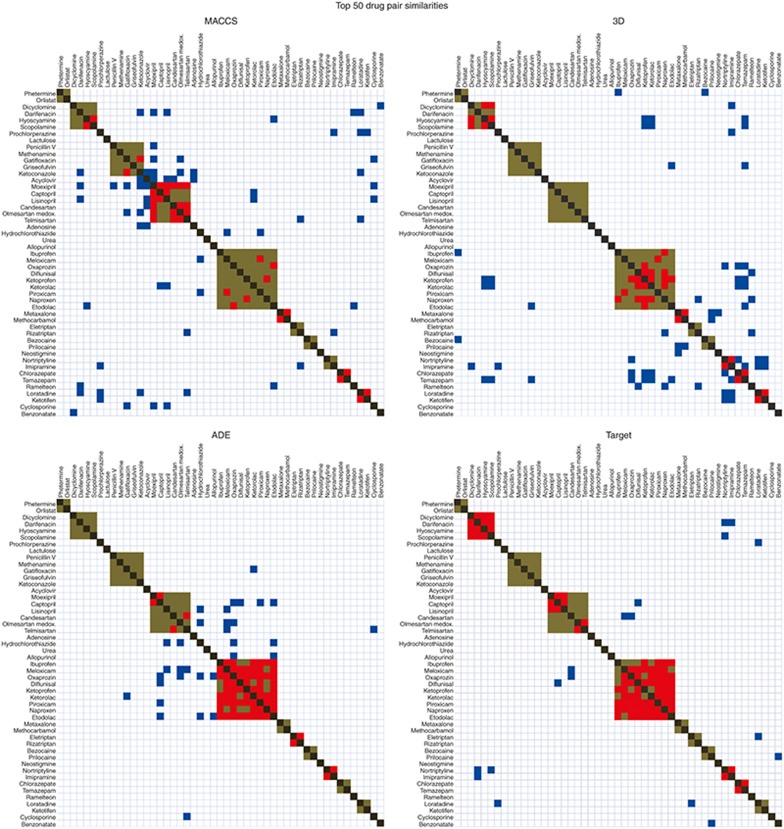
Similarity matrices, using 2D MACCS, 3D, ADE, and Target measures, for the set of drugs included in the ADE acute renal failure. We compared the different similarity methods to retrieve pharmacological classification results. In each matrix, the diagonal (dark gray) represents a drug against itself (in the plot, upper right and lower left are symmetric). Brown represents drug pairs belonging to the same pharmacological category and not retrieved in the top 50 similarity scores; red represents drug pairs belonging to the same pharmacological category and retrieved in the top 50 similarity scores; blue represents drug pairs belonging to different pharmacological category and retrieved in the top 50 similarity scores. Pharmacological categories range from well-defined classes, such as benzodiazepine anxiolytics, to broader classes, such as antibiotic–antifungals. The class with higher number of members is nonsteroidal anti-inflammatory drugs.

Similarity measures can show some degree of coincidence with the pharmacological class. To visualize this effect, we plotted in **Figure 4** the similarity matrix for the acute renal failure reference standard (positive and negative controls) using the four measures: 2D and 3D molecular structure and the knowledge-database ADE and Target similarities. Each matrix contains 49 columns and rows, the same number as the drugs in the reference standard (protein drugs in the initial reference standard, such as lipase, were not included). Drugs were grouped according to the pharmacological category (brown dots in the graphic). We represented in each matrix the top 50 drug pair similarity scorings. Red dots represent drug pairs retrieved in the top 50 belonging to the same pharmacological class. Blue dots represent drug pairs in which both drugs belong to different pharmacological category. In each matrix, there are 74 drug pairs belonging to the same pharmacological class within 1,176 possible drug pairs (=(49 × 49 − 49)/2). Random results would yield 3.15 drug pairs (=50 × 74/1,176) belonging to the same pharmacological class. As we expected, the bidimensional similarity matrices showed that all the similarity measures are related to the pharmacological classification showing results far from random (**Figure 4**; *P* < 0.001). However, the measures based on knowledge databases, such as ADE and Target similarities, are more related to the pharmacological classification than molecular structure measures. For the ADE and Target similarity matrices in the renal failure set, we retrieved 34 and 41 drug pairs (of 50) in the same pharmacological category, respectively (**Table 3**). However, in the case of 2D and 3D similarities, we retrieved 17 drug pairs in the same category in both cases. The same test has been conducted for the other ADE outcomes mentioned in the current article (**Table 3**). Results showed similar patterns in the comparison of the different similarity measures. The methods based on knowledge data detected above all similarity within the pharmacological class, whereas 2D and 3D molecular structure methods showed more flexibility to detect interclass similarity. This fact showed the pharmacological dependency of some knowledge data. ADE and Target similarities offered good results in the previous analysis improving the precision of the selected drug–ADE associations. However, the information provided by the similarity models in this case is obvious because the system detects preferably intraclass similarity. Otherwise, as **Figure 4** shows, 2D and 3D structural similarity have the potential of pointing out more challenging drug–ADE relationships. The figure demonstrates that the molecular structure (2D and 3D) and pharmacological class, although overlapping, are not identical and likely complementary.

## Discussion

Similarity methods were applied to medication-wide association studies performed in an administrative claims database with 46 million patients. However, similarity-based modeling can be applicable to improve signal detection steps using other data mining algorithms or other type of pharmacovigilance data, such as the FDA Adverse Event Reporting System or electronic health records.^21,22^ The method also allows rationalizing the relevance of the signals to optimize the decision-making process. However, these type of systems are not intended to replace other considerations used to evaluate the signal relevance, such as data consistency, biological relationships, or similar signals detected in other sources of data.^34^ Our intention is to provide additional information useful in the signal assessment. A more complete understanding of the conditions related to the ADE could improve drug patient safety processes.

In this study, similarity-based methods have been applied after the signals have been detected from healthcare databases with the intention of selecting final candidates pointed out by two different methodologies. However, implementation of similarity systems for all the drugs studied in healthcare databases would be an alternative to help in the early detection of ADEs related to newly marketed drugs with not enough accumulated exposure in the population.

The types of models showed in the current study are highly dependent on the training dataset, in our case, the ADE reference standard. Different structural and pharmacological complexity in the reference standard construction could be responsible for a different performance in the diverse ADE outcomes. For instance, 2D MACCS (Molecular Access System) model performs better in the outcomes renal and liver failure rather than myocardial infarction and GI ulcer. As an example, some drugs that cause myocardial infarction, such as frovatriptan, are not captured with good score when compared with other anti-migraine drugs also present in the reference standard, such as zolmitriptan (2D TC = 0.49). In fact, frovatriptan showed some differences in the molecular structure, such as a benzamide group not present in other triptan derivatives. Amlodipine and nifedipine, two calcium channel blockers, constitute another example of drugs with similar mechanism of action but not captured by our 2D similarity (TC = 0.48). Both drugs are derivatives of dihydropyridine, but different substitutions at molecular level makes difficult the similarity recognition according to MACCS fingerprint. On the other hand, the 3D model performs better in the ADEs myocardial infarction and GI ulcer than in renal and liver failure. The drugs mentioned above, frovatriptan, amlodipine, and nifedipine, showed a better score according to the 3D similarity (the 3D positive control score is 0.84, 0.82, and 0.82, respectively). As it was shown in previous studies,^35^ both 2D and 3D similarity measurements are complementary but different, capturing similarities between different pairs of drugs. Three-dimensional methods are capable of detecting connections between the structure and biological characteristics not captured by 2D methods and vice versa.

### Caveats and limitations

Performance of the similarity-based modeling is based on the reference standard database. External predictive power could be limited depending on the quality and comprehensiveness of the initial data. Additional improvements in the initial reference standard databases could be implemented for the construction of more reliable systems. When the reference standard reflects some complexity, i.e., drugs with similar pharmacological characteristics in both positive and negative controls, it would be advisable to use only similarity scorings against the positive control group to avoid cancellation of the similarity signal.

The nature of the similarity measurement is also an important limiting factor. Some fingerprints could contain certain bias information. As an example, ADE and Target fingerprints are calculated from the information contained in knowledge databases. Although these data sources are of great utility, the information available could be influenced by pharmacological classification of the drugs. Moreover, these types of similarity measures have difficulties to correctly evaluate similarity for some drugs for which there is scarce available information, i.e., this is the case of new drugs.

## Methodology

### Materials

*Healthcare data.* The database used to apply the similarity-based methods was collected from a previous publication.^20^ Analysis of four different ADEs was performed using an observational healthcare database: the CCAE administrative claims database.^2,12^ The database contained 46.5 million lives. Detailed description about the healthcare data has been published by our group previously.^20^

*Reference standard datasets.* Negative and positive controls, i.e., drugs that cause the ADEs or do not, were established based on natural language processing of structured product labels and systematic search of the scientific literature. We studied four clinically important ADEs: acute renal failure, acute liver failure, acute myocardial infarction, and upper GI ulcer. More details about drug reference standard data collection, including drug names, can be found in a previous publication.^20^ Protein drugs, such as lipase or darbepoetin alfa, were not included in our current databases when similarity measures are applied because molecular structure analysis is limited depending on size.

*Drug structure.* We collected the molecular structures of the drugs included in the study from DrugBank database.^29^ Structures of some drugs not available in DrugBank were downloaded from PubChem.^36^

### Methods

*SCCS analysis.* A SCCS analysis was performed for four different ADEs in the CCAE administrative claims database. A detailed description of the analysis has been published in the study by Ryan *et al*.^20,37,38^ Two sets of drug candidates for each ADE, using as thresholds *P* < 0.05 and *P* < 0.0005, were evaluated using similarity-based modeling as described below.

*Two-dimensional molecular structure similarity.* We calculated 2D molecular structure similarity between all the drugs in each database using a 2D molecular fingerprint called MACCS.^39,40^ In the fingerprint, we represent each drug as a bit vector that codifies in each position through 1 or 0 the presence or nonpresence of different structural keys. For instance, some structural keys represented in MACCS would be: position 11 codifies the presence of four-membered rings; position 78 codifies C=N groups; position 163 codifies six-membered rings, etc. To simplify the sparse binary vector, only the positions codifying the fragments that are present are retained in the final fingerprint representation. More details about MACCS fingerprint calculation are provided in a previous study.^22,41^

We used the Tanimoto coefficient (TC) to calculate the similarity between all drug pairs. The TC is a measurement between two fingerprints that ranges from 0 (minimum similarity) to 1 (maximum similarity). The TC is defined as:

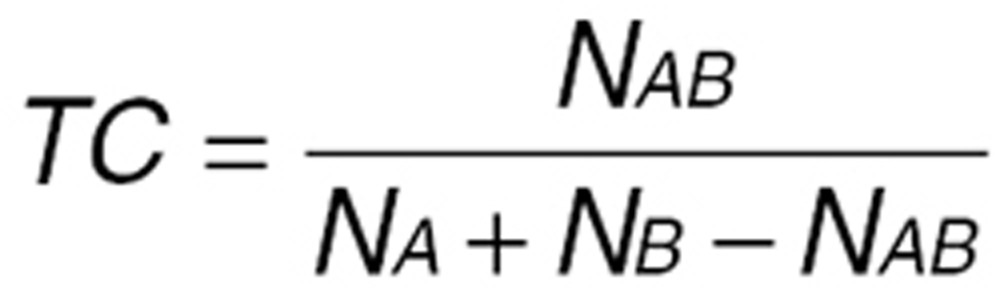


In the equation, *N*_AB_ is the number of features present in common in both fingerprints A and B, *N*_A_ is the number of features in fingerprint A, and *N*_B_ is the number of features in fingerprint B.

*Three-dimensional molecular structure similarity.* 3D similarity is calculated in different steps:

*1) Drug database preparation:* Bioactive conformations with specific chiral centers were downloaded from DrugBank.^29^ The drug database was prepared with LigPrep module from the Schrödinger 2011 package.^42^ We generated possible protonation states at neutral pH and a maximum of three enantiomers for unspecified chiral centers for some drugs. This process also involved optimization of the geometry of the structures using OPLS_2005,^43^ the force field that describes the potential energy of the system.

*2) Conformational analysis:* The 3D structure of each drug in the databases was determined through conformational analysis using water as an implicit solvent in the Macromodel module from Schrödinger.^44^ The engine search method was Monte Carlo Multiple Minimum. For simplicity reasons, only the structure with the global minimum potential energy OPLS_2005 was retained and used as a template in the shape screening step.

*3) Shape screening calculations:* We used the 3D structures determined in the previous conformational analysis as template queries to run shape screening calculations for all the drugs included in the study using phase module from Schrödinger package.^45^ Our objective is to identify similar shape and pharmacophoric properties between all the drugs. The calculation generated a maximum of 500 conformers for each drug and aligned them to each template. We calculated a 3D similarity score between all the drug pairs that ranges from 0 to 1. The similarity score, called Phase Sim property, was calculated considering the overlapping volume between atoms that present the same pharmacophoric characteristics.^45^

*ADE similarity:* The data about adverse effects were downloaded from SIDER database.^46^ SIDER is a side effect resource that contains information about 996 medicines and 4,192 adverse effects extracted from public documents and package inserts. Although SIDER is a useful source, not all the drug reactions are totally confirmed, and in some cases, further studies would be necessary. However, for the four ADE outcomes, SIDER database showed a high degree of coincidence with our reference standard: 94, 84, 92, and 97% of coincidence between SIDER and our reference standards for acute renal failure, acute liver failure, acute myocardial infarction, and upper GI ulcer, respectively. For labeling the drugs in SIDER as positive or negative, we used the specified ADE terms and related (example for renal failure: renal failure acute, acute renal insufficiency, shutdown renal, etc).

We calculated ADE similarity through the use of fingerprints. For each of the four studied ADEs, we excluded in SIDER the ADE itself and related terms in the calculation of the fingerprint. The concept of the ADE fingerprint is similar to 2D molecular structure fingerprints. In the ADE fingerprints, we codified in the different bit positions the presence or absence (code 1 or 0, respectively) of different adverse events associated with the drugs. As an example, some adverse events represented in the calculation of ADE fingerprints would be: position 1 codifies the presence of the ADE abasia; position 82 codifies the presence of the ADE acidosis; position 563 codifies the presence of the ADE bullous eruption; position 3,385 codifies the presence of the ADE rhabdomyolysis, etc. We retained in the final ADE fingerprint only the positions codifying the fragments that are present (sparse binary vector simplification). As described above (see Methods section for 2D molecular structure similarity), we calculated the TC between all the drug pair fingerprints.

*Target similarity:* Data about drug targets were downloaded from DrugBank database.^29^ We integrated the drug targets database with the enzymes, transporters, and carriers datasets from DrugBank. Repeated cases were eliminated. We also considered targets from different species/organisms as a unique target case. The procedure to calculate the Target fingerprints is the same as described before in ADE fingerprints or MACCS, but instead of considering adverse events or the structural keys, we listed now targets for each bit vector position. Therefore, we calculated TC between all the Target fingerprints.

*ATC similarity:* The ATC Classification System^47^ is used for the classification of drugs. Chemical characteristics, therapeutic action, and information about the system or organ on which the drugs act are introduced in the database. Because some drugs could have several classification codes, a manual revision of the classification was made to provide more accurate data. The different categories in the database are codified in bit positions of a fingerprint as explained previously. We used the TC to calculate similarity between all the ATC fingerprints.

*Construction of ADE similarity matrix and scoring extraction:* All drug pair similarities, based on TC or Phase Sim property, for each ADE reference standard database were integrated in a similarity matrix. A score for each drug in the ADE reference standard is calculated through a leave-one-out procedure. Each drug was taken out and evaluated by the similarity model to compare the performance with the rest of the candidate drugs. For every drug is defined a maximum pairwise score (TC or Phase Sim property) obtained against each drug that produces the ADE in the reference standard dataset. A second pairwise maximum similarity score against the set of drugs that do not produce the ADE is calculated. The final score is provided by the difference between similarity scores, the ADE score, and the non-ADE score. As an example, the drug meloxicam in the acute renal failure reference standard was compared in the 2D MACCS model against the set of drugs that produce the ADE, and the maximum TC pairwise was 0.89. On the other hand, the maximum TC against the non-ADE drugs was 0.49. The final difference scoring was 0.40.

*Assessment of the similarity models:* Evaluation of the similarity methods applied to the four sets of ADE candidates was compared with the results ranking the drug candidates using *P* values and RR. The evaluation focused on the proportion of true positives for each ADE identified by every approach. We calculated the precision of the methods (TP/TP+FP) as a standard measurement to compare the performance. Precision-Recall graphics were plotted for the four ADEs considering as true positives and false positives the drugs in the reference standard deemed as positive and negative controls, respectively. Areas under the ROC curves were also reported to compare the performance of the different methods within the *P* < 0.05 selected subset of candidates (pAUROC). We also evaluated through ROC curves the global performance of the SCCS analysis in the CCAE administrative claims database.

## Author Contributions

S.V., P.R., N.P.T., and G.H. wrote the manuscript. S.V. and G.H. designed the research. S.V. and P.R. performed the research. S.V. analyzed the data. S.V., P.R., D.M., P.S., M.S., C.F., N.P.T., and G.H. contributed new reagents/analytical tools.

## Conflict of Interest

The authors declared no conflict of interest.

## Study Highlights


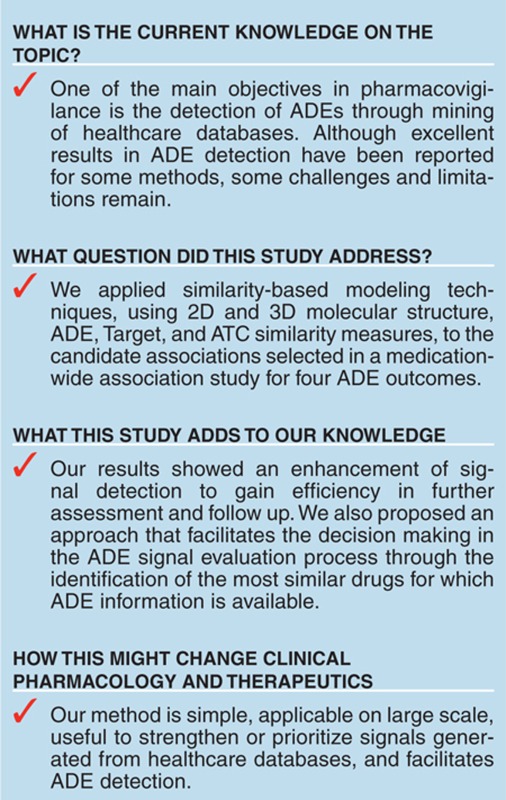


## Figures and Tables

**Figure 1:**
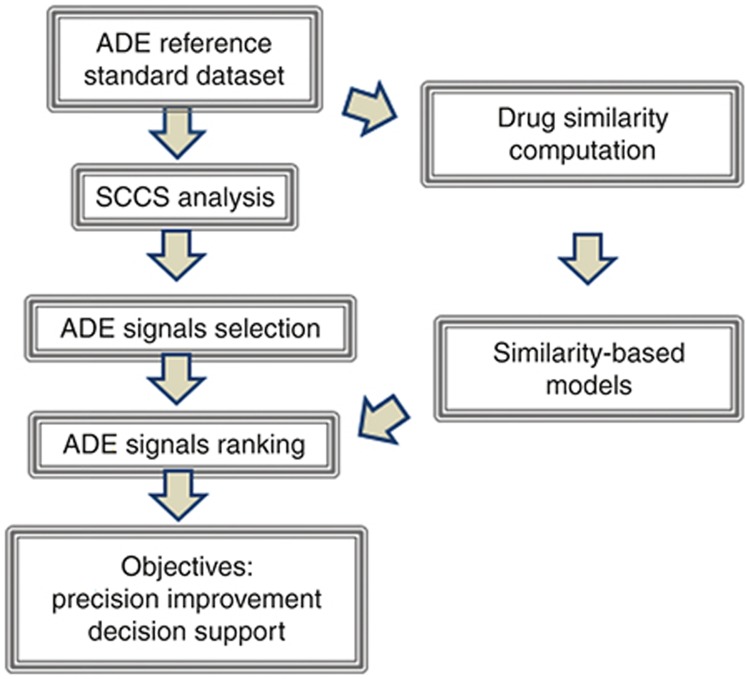
Flowchart of the different steps implicated in the current analysis.

**Table 1:**
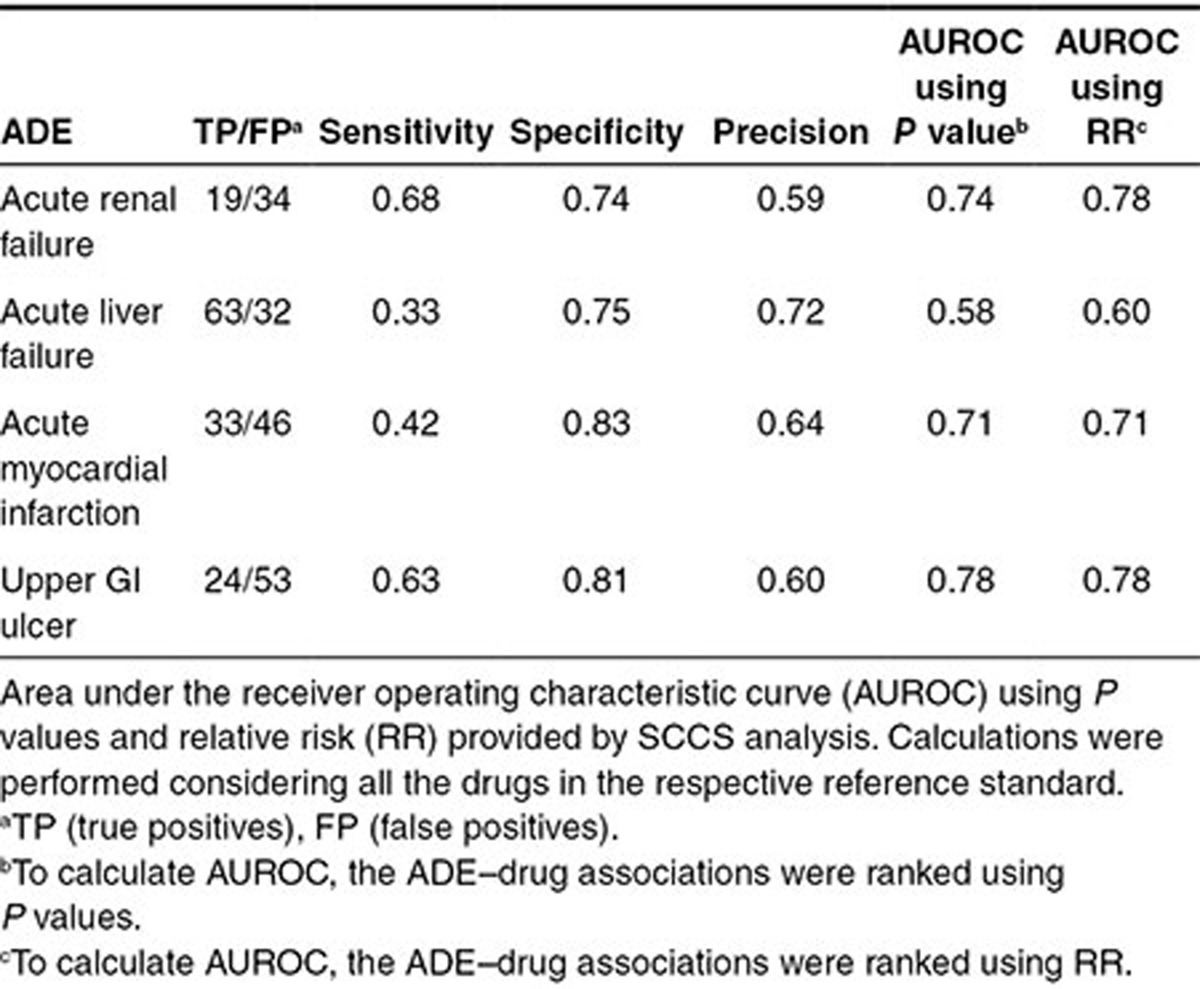
Results provided by SCCS analysis in the Truven MarketScan CCAE administrative claims database for four ADEs

**Table 2:**
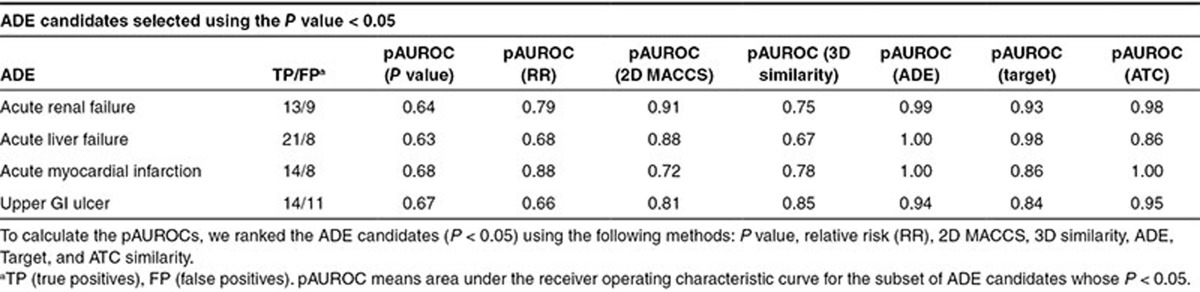
pAUROC results for the subset of ADE candidates whose *P* values extracted from SCCS analysis are <0.05

**Table 3:**
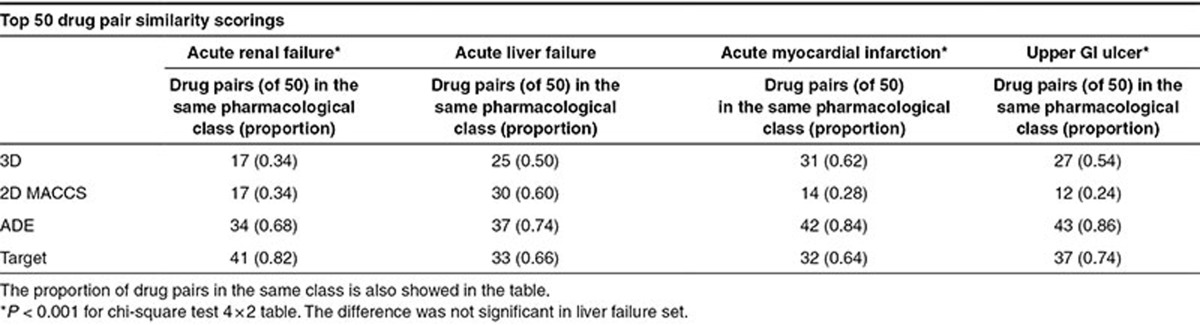
Number of drug pairs that belong to the same pharmacological class retrieved by the different similarity measures within the top 50 scores
