# Supplementary figures and images for: Similarity-Based Modeling Applied to Signal Detection in Pharmacovigilance
Source: CPT Pharmacometrics Syst Pharmacol. 2014 Sep 24;3(9):e137–. doi: 10.1038/psp.2014.35 (PMC4211266; doi:10.1038/psp.2014.35)

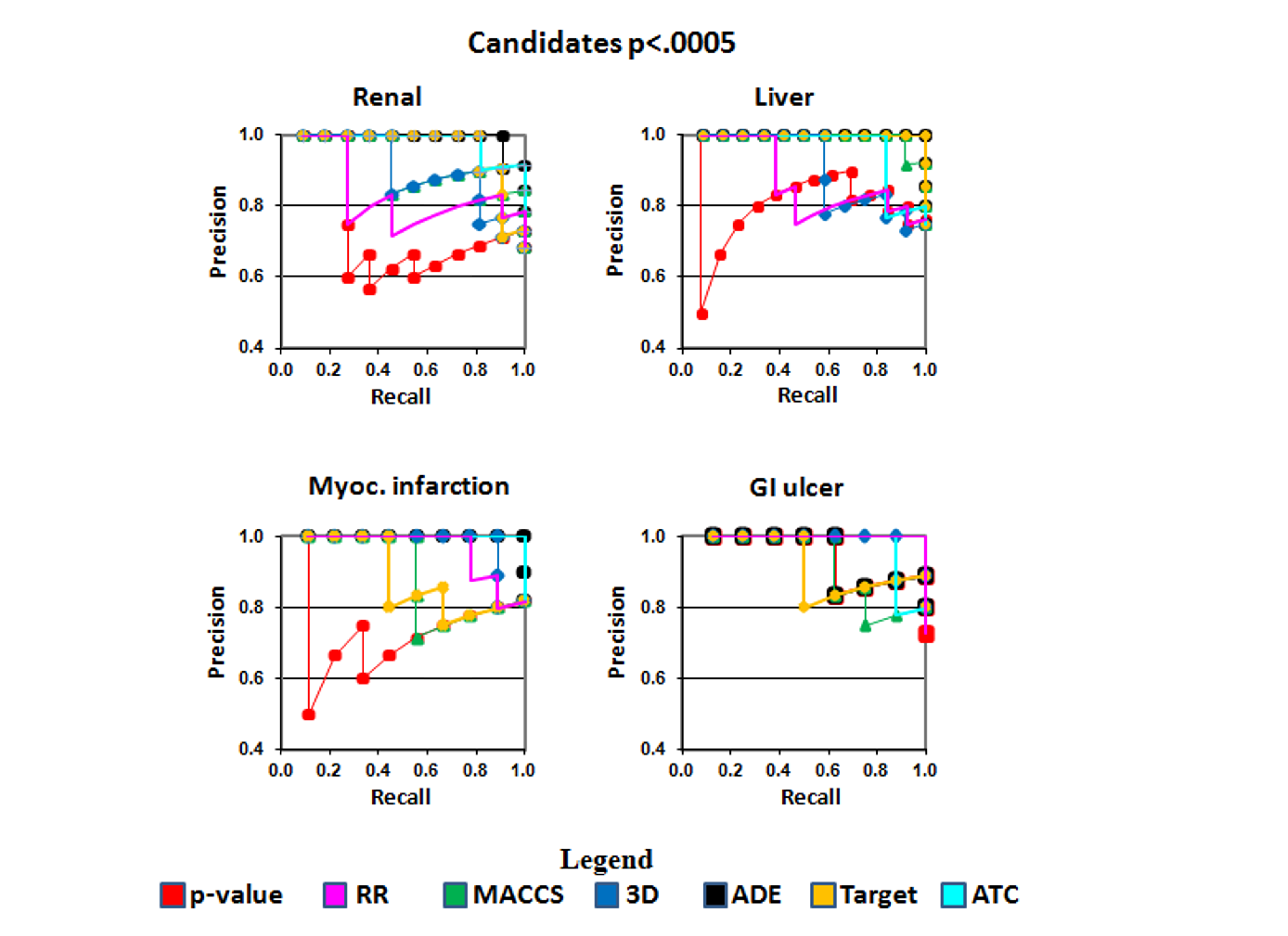

Supplement: Supplementary Figure S1 [file psp201435x1.tiff]
