# Supplementary material for: Similarity-Based Modeling Applied to Signal Detection in Pharmacovigilance
Source: CPT Pharmacometrics Syst Pharmacol. 2014 Sep 24;3(9):e137–. doi: 10.1038/psp.2014.35 (PMC4211266; doi:10.1038/psp.2014.35)
Supplement: Supplementary Table S1 [file psp201435x2.doc]

**Supplementary Material**

**Table S1.** Precision in the ADE candidate sets in different top positions using different methods to sort the drug candidates (methods: *p*-value, Relative Risk (RR), 2D MACCS, 3D similarity, ADE, Target and ATC similarity). For each ADE two sets were selected based on *p*-value cut-offs.

| **TOP position** | ***p*-value** |  | **RR** | **2D MACCS** | **3D similarity** | **ADE** | **Target** | **ATC** |
| --- | --- | --- | --- | --- | --- | --- | --- | --- |
| **Acute renal failure (*p*<.05)** | | | | | | | | |
| 5 | 0.60 |  | 0.80 | 1.00 | 0.80 | 1.00 | 1.00 | 1.00 |
| 10 | 0.60 |  | 0.80 | 0.90 | 0.80 | 1.00 | 1.00 | 1.00 |
| 15 | 0.73 |  | 0.80 | 0.87 | 0.73 | 0.87 | 0.80 | 0.87 |
| 20 | 0.60 |  | 0.60 | 0.65 | 0.60 | 0.65 | 0.65 | 0.65 |
| **Acute renal failure (*p*<.0005)** | | | | | | | | |
| 5 | 0.60 |  | 0.80 | 1.00 | 1.00 | 1.00 | 1.00 | 1.00 |
| 10 | 0.60 |  | 0.80 | 0.90 | 0.90 | 1.00 | 0.90 | 0.90 |
| 15 | 0.73 |  | 0.73 | 0.73 | 0.73 | 0.73 | 0.73 | 0.73 |
|  |  | | | | | | |  |
| **Acute liver failure (*p*<.05)** | | | | | | | | |
| 5 | 0.80 |  | 1.00 | 0.80 | 0.80 | 1.00 | 1.00 | 1.00 |
| 10 | 0.90 |  | 0.80 | 0.90 | 0.90 | 1.00 | 1.00 | 1.00 |
| 15 | 0.80 |  | 0.80 | 0.93 | 0.80 | 1.00 | 1.00 | 0.93 |
| 20 | 0.75 |  | 0.75 | 0.95 | 0.80 | 1.00 | 0.95 | 0.90 |
| 25 | 0.76 |  | 0.76 | 0.80 | 0.76 | 0.80 | 0.80 | 0.76 |
| **Acute liver failure (*p*<.0005)** | | | | | | | | |
| 5 | 0.80 |  | 1.00 | 1.00 | 1.00 | 1.00 | 1.00 | 1.00 |
| 10 | 0.90 |  | 0.80 | 1.00 | 0.80 | 1.00 | 1.00 | 1.00 |
| 15 | 0.80 |  | 0.80 | 0.80 | 0.73 | 0.80 | 0.80 | 0.80 |
|  |  | | | | | | |  |
| **Acute myocardial infarction (*p*<.05)** | | | | | | | | |
| 5 | 0.60 |  | 1.00 | 1.00 | 1.00 | 1.00 | 1.00 | 1.00 |
| 10 | 0.80 |  | 0.90 | 0.80 | 0.80 | 1.00 | 0.90 | 1.00 |
| 15 | 0.73 |  | 0.80 | 0.67 | 0.73 | 0.93 | 0.80 | 0.93 |
| 20 | 0.65 |  | 0.70 | 0.65 | 0.70 | 0.70 | 0.70 | 0.70 |
| **Acute myocardial infarction (*p*<.0005)** | | | | | | | | |
| 5 | 0.60 |  | 1.00 | 1.00 | 1.00 | 1.00 | 0.80 | 1.00 |
| 10 | 0.80 |  | 0.80 | 0.80 | 0.80 | 0.90 | 0.80 | 0.90 |
|  |  | | | | | | |  |
| **Upper gastrointestinal ulcer (*p*<.05)** | | | | | | | | |
| 5 | 1.00 |  | 0.80 | 1.00 | 1.00 | 1.00 | 1.00 | 1.00 |
| 10 | 0.80 |  | 0.70 | 0.80 | 0.80 | 1.00 | 0.90 | 1.00 |
| 15 | 0.53 |  | 0.73 | 0.73 | 0.80 | 0.80 | 0.73 | 0.87 |
| 20 | 0.60 |  | 0.60 | 0.65 | 0.70 | 0.70 | 0.65 | 0.65 |
| **Upper gastrointestinal ulcer (*p*<.0005)** | | | | | | | | |
| 5 | 1.00 |  | 1.00 | 1.00 | 1.00 | 1.00 | 0.80 | 1.00 |
| 10 | 0.80 |  | 0.80 | 0.80 | 0.80 | 0.80 | 0.80 | 0.80 |
